# Tremor Reduction by Deep Brain Stimulation Is Associated With Gamma Power Suppression in Parkinson's Disease

**DOI:** 10.1111/ner.12297

**Published:** 2015-04-16

**Authors:** Martijn Beudel, Simon Little, Alek Pogosyan, Keyoumars Ashkan, Thomas Foltynie, Patricia Limousin, Ludvic Zrinzo, Marwan Hariz, Marko Bogdanovic, Binith Cheeran, Alexander L. Green, Tipu Aziz, Wesley Thevathasan, Peter Brown

**Affiliations:** ^1^ Nuffield Department of Clinical Neurosciences, John Radcliffe HospitalUniversity of OxfordOxfordUK; ^2^ Department of Neurology, University Medical Centre GroningenUniversity of GroningenGroningenThe Netherlands; ^3^ Department of Neurosurgery, Kings College HospitalKings College LondonLondonUK; ^4^ Unit of Functional Neurosurgery, Sobell Department of Motor Neuroscience & Movement DisordersUCL Institute of Neurology, Queen SquareLondonUK; ^5^ Melbourne Brain Centre, Department of Medicine, Royal Melbourne HospitalUniversity of MelbourneMelbourneVictoriaAustralia; ^6^ The Bionics InstituteMelbourneVictoriaAustralia

**Keywords:** Deep brain stimulation, mechanisms of action, Parkinson's disease, subthalamic nucleus, tremor

## Abstract

**Objectives:**

Rest tremor is a cardinal symptom of Parkinson's disease (PD), and is readily suppressed by deep brain stimulation (DBS) of the subthalamic nucleus (STN). The therapeutic effect of the latter on bradykinesia and rigidity has been associated with the suppression of exaggerated beta (13–30 Hz) band synchronization in the vicinity of the stimulating electrode, but there is no correlation between beta suppression and tremor amplitude. In the present study, we investigate whether tremor suppression is related to suppression of activities at other frequencies.

**Materials and Methods:**

We recorded hand tremor and contralateral local field potential (LFP) activity from DBS electrodes during stimulation of the STN in 15 hemispheres in 11 patients with PD. DBS was applied with increasing voltages starting at 0.5 V until tremor suppression was achieved or until 4.5 V was reached.

**Results:**

Tremor was reduced to 48.9% ± 10.9% of that without DBS once stimulation reached 2.5–3 V (t14 = −4.667, *p* < 0.001). There was a parallel suppression of low gamma (31–45 Hz) power to 92.5% ± 3% (t14 = −2.348, *p* = 0.034). This was not seen over a band containing tremor frequencies and their harmonic (4–12 Hz), or over the beta band. Moreover, low gamma power correlated with tremor severity (mean *r* = 0.43 ± 0.14, *p* = 0.008) within subjects. This was not the case for LFP power in the other two bands.

**Conclusions:**

Our findings support a relationship between low gamma oscillations and PD tremor, and reinforce the principle that the subthalamic LFP is a rich signal that may contain information about the severity of multiple different Parkinsonian features.

## Introduction

Resting tremor is, in conjunction with bradykinesia, rigidity, and postural instability, one of the four cardinal symptoms of Parkinson's disease (PD) 1. Rest tremor affects ∼70% of patients and occurs at 4 to 6 Hz 2. Although the pathological hallmark of PD is well established, namely progressive degeneration of midbrain dopaminergic neurons and their projections to the basal ganglia, and the striatum in particular 3, it is not clear how this relates to resting tremor. Recent analyses suggest that resting tremor may have a distinct underlying pathophysiology to that of bradykinesia and rigidity 4. This is in line with earlier findings indicating different pathological changes in tremor dominant and akinetic‐rigid PD patients 5. The dichotomy is further emphasized by the finding that the suppression of beta power in the subthalamic nucleus (STN) upon treatment with levodopa or deep brain stimulation (DBS) correlates with improvements in bradykinesia and rigidity, but not with those in tremor 6, 7, 8, 9. In contrast, depth recordings in PD point to an association between low gamma oscillations in the STN and tremor severity 10. The question we address here is whether changes in low gamma activity correlate with tremor suppression during DBS. A positive outcome would strengthen the argument that the level of gamma oscillations in the STN might be related to tremor.

## Materials and Methods

### Patients and Recordings

We studied 11 patients (four women) with advanced idiopathic PD undergoing DBS electrode implantation in the STN (Table 1). This was a multicenter study in which patients were recruited in either Oxford (John Radcliffe Hospital) or London (National Hospital for Neurology and Neurosurgery or Kings' College Hospital); centers that have previously successfully pooled electrophysiological data 10, 11, 12, 13. All patients gave their informed written consent for the study, which was approved by the local ethics committee. All patients had tremor pre‐ and postsurgery, but were not necessarily tremor dominant. Patients underwent surgery in a two‐stage procedure with bilateral quadripolar electrodes (model 3389, Medtronic Neurologic Division, Minneapolis, MN, USA). The electrode placement and stimulator implantation were separated by one week, as previously described 14. All testing was performed two to six days after electrode implantation.

**Table 1:** Clinical and Stimulation Details of Patients

	Patient 1	Patient 2	Patient 3	Patient 4	Patient 5	Patient 6	Patient 7	Patient 8	Patient 9	Patient 10	Patient 11	Mean + SEM
Age (years)	59	59	48	54	62	78	66	61	51	52	70	60 ± 2.7
Disease duration (years)	14	16	20	9	9	5	6	13	11	8	13	11.2 ± 1.3
UPDRS off‐drugs	59	42	56	19	46	29	32	33	38	40	45	39.9 ± 3.5
UPDRS on‐drugs	19	10	33	6	16	26	4	12	13	11	35	16.8 ± 3.1
Surgery location	OX	OX	OX	OX	OX	UCL	OX	UCL	UCL	UCL	KC	—
Initial symptom	Tremor	Tremor	Rigidity	Tremor	Rigidity	Tremor	Tremor	Tremor	Tremor	Tremor	Tremor	—
Primary DBS indication	Tremor	Tremor	Off‐periods	Off‐periods	Off‐periods	Tremor	Off‐periods	Tremor	Off‐periods	Off‐periods	Tremor	—
Preoperative drugs	Levodopa, Pramipexole	Levodopa, Amantadine, Cabergoline	Levodopa, Ropinirole	Levodopa, Entacapone, Pramipexole, Selegeline	Levodopa, Tolcapone	Levodopa, Entacapone	Levodopa, Pramipexole	Levodopa, Ropinirole, Rasagiline, Amantadine	Levodopa, Entacapone, Rotigotine	Levodopa, Rotigotine	Levodopa, Rotigotine	—
LED (mg/day)	1080	1050	700	1700	800	1200	1315	1715	1573	620	1140	1172 ± 114
Recorded hemisphere(s)	L/R	L	R	L/R	L/R	L	L	R	L	L/R	R	—
50% tremor suppression voltage	1.5/0.5	1	3.0	1/2.5	3/3.5	3.5	3.5	2.5	2.5	1.5	2	2.3 ± 0.3

Fifty percent tremor suppression voltages indicate the stimulation voltage at which tremor was reduced to 50% of its original amplitude.

DBS, deep brain stimulation; KC, King's College; L, left; LED, levodopa equivalent dose; OX, Oxford; R, right; SEM, standard error of the mean; UCL, University College London; UPDRS, Unified Parkinson's Disease Rating Scale Part III (motor).

We recorded bipolar local field potential (LFP) activity from the STN electrode contacts after ovenight withdrawal of anti‐parkinsonian medication. In subjects with bilateral upper limb tremor, bilateral recordings were performed. For unilateral upper limb tremor, only the hemisphere contralateral to the affected side was recorded. In all, we recorded 15 hemispheres (including four bilateral studies). Tremor was also recorded using a tri‐axial accelerometer (Twente Medical Systems International, Oldenzaal, The Netherlands) taped to the dorsal surface of the hand while patients sat with the hand comfortably on their lap in a semi‐pronated position.

To overcome stimulation artifacts, LFP recordings were made through a single‐channel, isolated, two‐stage, high‐gain (100 dB) amplifier with narrow pass band to record bipolar LFP signals from electrode contacts 0 and 2 or 1 and 3 while the contact in the middle (contact 1 or contact 2, respectively) was stimulated. Rossi et al. initially reported this approach 15. It has been subsequently further developed, extensively tested and applied 16. Supplementary fig. S2A in the latter reference demonstrates that the amplifier pass band was relatively flat between 5 and 45 Hz, with ≤20% signal amplitude fall off at the margins of this pass band. Bipolar recordings were made from the contact pair (0–2 or 1–3) exhibiting the highest beta amplitude and were determined for each hemisphere independently in the unstimulated OFF‐medication state. We recorded from the contact pair that afforded the highest beta power as this activity is reported to originate in the dorsal ‘motor’ STN 7, 17. Tremor and LFP data were sampled at 2048 Hz and recorded through a 1401 A‐D converter (Cambridge Electronic Designs, Cambridge, UK) onto a personal computer running Spike2 software (Cambridge Electronic Designs). Stimulation was performed in a monopolar fashion through the contact (1 or 2) between the two recording contacts. Stimulation was applied unilaterally at 130 Hz with a pulse width of 90 μsec using a DualStim external stimulator (Medtronic Neurologic Division). Stimulation started at 0.5 V and was increased in 0.5 V increments at 100 s intervals until either complete tremor suppression was achieved or 4.5 V was reached.

### Data Analysis

After visual inspection, LFPs and tri‐axial accelerometer signals were analyzed offline using custom‐written scripts in MATLAB (version 7.10; MathWorks, Natick, MA, USA). The accelerometer axis that recorded the greatest tremor amplitude was selected for further analysis. Both the LFP and selected accelerometer data were divided into epochs of 100 sec in which each epoch represented stimulation with a certain fixed voltage (0–max 4.5 V). The amplitudes of both the tremor signal and the LFP were calculated using a Morlet wavelet transformation. For the tremor signal, this was centered on the peak frequency ±1 Hz, and for the LFP this was calculated for all frequencies between 1 and 45 Hz before being averaged across the bands of interest. The amplitudes for all points were then averaged over each stimulation block to give a single value of amplitude for the tremor and LFP in each frequency band for each level of DBS stimulation. These data were then taken forward for further analysis. Spectral decomposition with a continuous wavelet transform was performed because of its efficient time frequency resolution and lack of assumptions regarding stationarity 18, 19.

The three contiguous frequency ranges were selected as: 4–12 Hz, 13–30 Hz, and 31–45 Hz. The 4–12 Hz range was chosen to correspond to tremor frequency and its harmonic, as both have been identified in brain recordings of tremulous patients 20, 21. The 13–30 Hz range was chosen to correspond to the beta activity which is regularly reported in STN recordings 6, 9, and the 31–45 Hz range selected to sample low gamma activity (up to the limit of the low pass filter), as changes in this approximate frequency band have been previously implicated in PD rest tremor 22.

For group analyses, accelerometer and LFP data in the different spectra were normalized to the initial value without stimulation (0 V) and expressed as a percentage of the initial value. Data were described using means and standard error of means (SEM), and normality was confirmed using the Kolmogorov–Smirnov test (*p* > 0.05).

Changes in tremor amplitude and LFP band power were assessed in separate repeated measures ANOVAs during the 0.5–1 V, 1.5–2 V, and 2.5–3 V ranges of stimulation and Bonferroni correction applied. The unstimulated baseline was not used as, with normalization, this had a value of 1. Where more than one value was available for each hemisphere in a given stimulation range (e.g., 0.5 and 1 V), these were averaged. In one subject, tremor was totally suppressed at 2.0 V and stimulation therefore not performed at higher intensities. Accordingly, in this subject, values for 1.5–2V were conservatively repeated for 2.5–3V. Where Greenhouse–Geisser corrections were applied, corrected degrees of freedom are cited. To examine the relationship between tremor amplitude and LFP band power within hemispheres, correlation analyses were performed using Spearman's correlation. Correlation coefficients were normally distributed and their difference from zero tested using a one‐sample *t*‐test.

## Results

### Suppression of Tremor and LFP Activities

**Figure 1:**
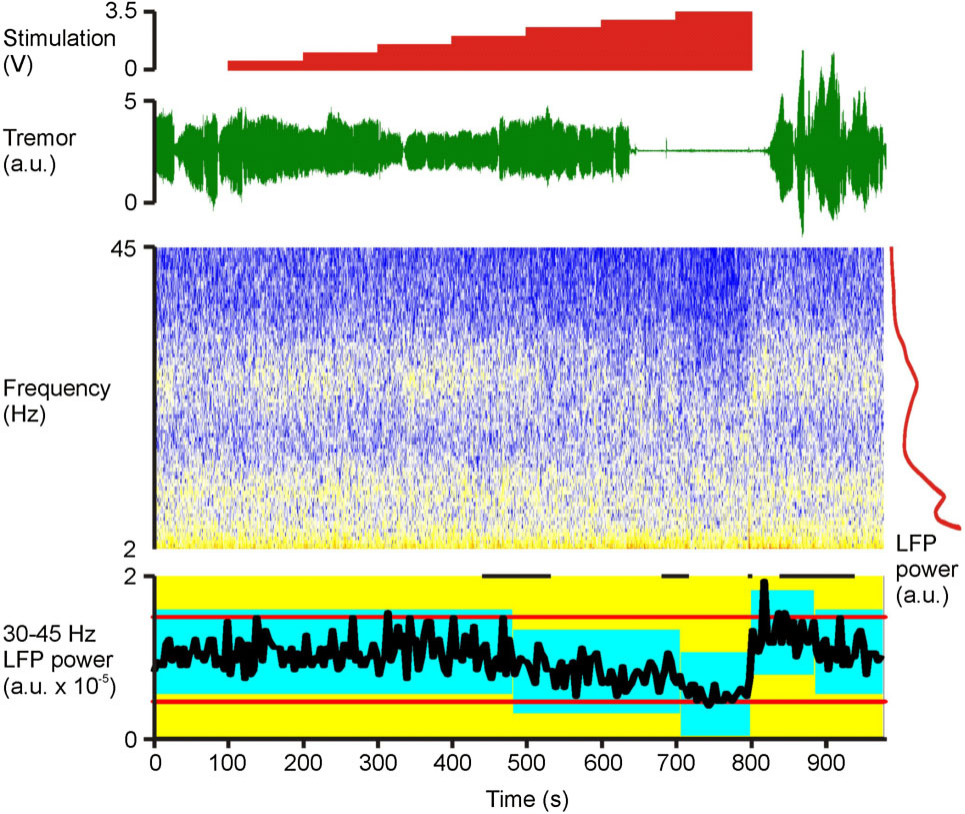
Example recording (case 3). Time‐evolving STN LFP spectrum and contralateral upper limb tremor are shown, together with the timings of incremental changes in stimulation. No stimulation is applied at the beginning and end of the recordings. The LFP is shown as a spectrogram, with cold colors representing lowest power (so blue, white, yellow, and then red reflect increasing power). To the right of time‐evolving spectrogram is a time‐averaged LFP power spectrum in red demonstrating discrete peaks at twice tremor frequency (10 Hz) and in the beta band (peaking at 26 Hz), but no such discrete peak in the low gamma (31–45 Hz) band. Onset and offset of tremor suppression is delayed for a few seconds after corresponding voltage changes. In contrast, return of gamma power to baseline levels is very rapid upon cessation of stimulation. Below the time‐evolving spectrogram (bottom trace) is the control chart of the 31–45 Hz LFP power estimated in nonoverlapping 4 sec blocks. Red horizontal lines either side of the power trace in black are the control limits of the whole recording. The blue blocks represent periods of constant 31–45 Hz power identified by change‐point analysis (*p* < 0.01) 23. The vertical extent of the blue blocks denotes the 99% confidence limits centered on the mean of each stable period. Short black horizontal lines at the top are the 99% confidence limits of the changes in gamma power (10,000 bootstraps). Change‐point analysis independently confirms that gamma power was reduced around the time of increasing stimulation voltage to 2.5 V and 3.5 V. Gamma power then rebounded when stimulation was stopped. Stimulation was delivered at 130 Hz with 90 μsec pulse width and applied at contact 1. The LFP was recorded from contacts 02 on the right. The tremor was recorded with an accelerometer taped to the dorsum of the left (contralateral) hand. LFP, local field potential; STN, subthalamic nucleus.

**Figure 2:**
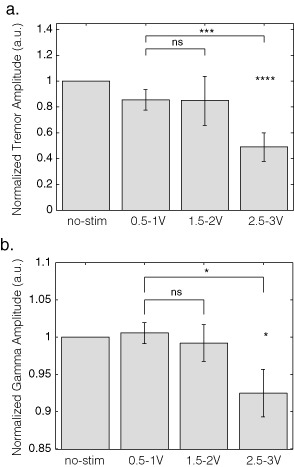
Average amplitudes of (a) tremor and (b) 31–45 Hz activities over the different stimulation ranges. Data are normalized to the no‐stimulation condition (left column). Vertical gray bars depict mean values and the black stripes the standard error of this mean. a.u., arbitrary units; no‐stim, no‐stimulation; ns, nonsignificant. *p < 0.05; ***p < 0.005; ****p < 0.001.

Figure 1 shows a representative example of changes in tremor and STN LFP during stimulation at incrementally increased voltages. Note that both tremor amplitude and low gamma (31–45 Hz) LFP activity are suppressed at higher stimulation voltages, although suppression of tremor seems to lag behind both the voltage and gamma changes by a few seconds and re‐emerges later than gamma power after stimulation had stopped. None of the subjects demonstrated a discrete peak in the low gamma band. Repeated measures ANOVA demonstrated a main effect of stimulation on tremor amplitude (*F*_2,28_ = 4.909, *p* = 0.015). Simple within‐subject contrasts indicated no difference between 0.5–1 V and 1.5–2 V (T_1,14_ = 0.001, *p* = 0.970), but did demonstrate a significant difference between 0.5–1 V and 2.5–3 V (T_1,14_ = 14.965, *p* = 0.002). During stimulation at 2.5–3V, tremor was decreased to 48.9% ± 10.9% compared with the no stimulation condition across all subjects (T_14_ = −4.667, *p* < 0.001, one‐sample *t*‐test; Fig. 2a). LFP amplitude (31–45 Hz) was found to be significantly reduced during stimulation (*F*_2,28_ = 6.015, *p* = 0.007, ANOVA; Fig. 1b). Simple within‐subject contrasts indicated no difference between 0.5–1 V and 1.5–2 V (T_1,14_ = 0.474, *p* = 0.502), but a difference between 0.5–1 V and 2.5–3 V (T_1,14_ = 8.392, *p*= 0.012). During stimulation at 2.5–3 V, 31–45 Hz LFP amplitude was suppressed to 92.5% ± 3% compared with the no stimulation condition (T_14_ = −2.348, *p* = 0.034, one‐sample *t*‐test; Fig. 2b). ANOVAs assessing 4–12 Hz and 13–30 Hz LFP amplitudes over the different stimulation voltages were not significant after Bonferroni correction for multiple ANOVAs (*F*_2,28_ = 1.27, *p* = 0.292 and *F*_1.388,19.426_ = 4.695, *p* = 0.032, respectively, with a Bonferroni corrected significant *p* value of 0.0125).

### Correlation Between Tremor and LFP Activities

**Figure 3:**
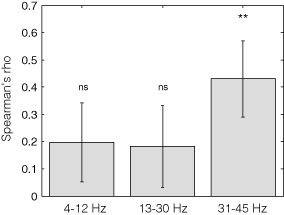
Average correlations of 4–12, 13–30, and 31–45 Hz band LFP and tremor amplitudes. Vertical bars depict the mean Spearman's rho of the correlation analyses between normalized tremor amplitude and normalized 4–12, 13–30, and 31–45 Hz power. The black stripes indicate the standard error of the mean. ns, nonsignificant. **p < 0.01. LFP, local field potential.

Within subjects, 31–45 Hz LFP amplitude across stimulation blocks correlated positively with tremor amplitude in 12 of the 15 hemispheres (and in seven, this was individually significant). The average Spearman's correlation coefficient was 0.43 ± 0.14 which was found to be significantly different from 0 (T_14_ = 3.07, *p* = 0.008; less than the Bonferroni corrected *p* value of 0.017, one‐sample *t*‐test, Fig. 3). The 13–30 Hz LFP amplitude showed only a weak correlation with tremor amplitude. The average correlation coefficient was 0.18 ± 0.15 and did not differ significantly from 0 (T_14_ = 1.21, *p* = 0.24, Fig. 3). The 4–12 Hz amplitude also only showed a weak correlation with tremor amplitude with an average correlation coefficient of 0.19 ± 0.14 that did not differ significantly from 0 (T_14_ = 1.35, *p* = 0.19, Fig. 3).

## Discussion

We have shown that DBS induced suppression of low gamma LFP power in the STN correlates with DBS‐induced reductions in the amplitude of resting tremor. These correlations were frequency selective and not shared by slower LFP activities. In particular, it is striking that correlations were absent from the LFP in the 4–12 Hz band, despite the fact that tremor‐related oscillations are common in single‐unit recordings 22, 24, 25. However, the variable phase relationships between neurons oscillating at tremor frequencies mean that this feature is not well represented in the LFP 22, 24, 26.

The present study contains several limitations. First, in order to recruit a sufficiently large patient sample, we studied patients at several different surgical centers, which may have introduced variance related to the DBS positioning strategy in our dataset. However, although the numbers are too low to apply statistics, we did not see differences in either baseline characteristics or tremor suppression characteristics between the different centers. Second, our study does not address the question of the precise site of origin of the low gamma activity. We recorded from the contact pair that afforded the highest beta power, given that this activity is supposed to originate in the dorsal “motor” STN 7, 17, but in order to harness common‐mode rejection by our amplifier during DBS we were limited to recordings from rather widely spaced contact pairs. Nevertheless, Weinberger et al. 22 have shown that the increase in gamma activity during tremor is most likely to occur at sites in the dorsal STN. Third, recordings were performed only a few days after electrode implantation, when temporary stun effects may be present, leading to increased electrode impedances, with potential changes in recorded signal voltages, and temporary improvement in baseline impairments 27, 28, 29, 30. Future studies using the next generation of implantable of impulse generators with recording facilities are necessary to confirm whether the findings reported here remain representative during chronic stimulation, when any potential stun effects have elapsed 31. Finally, there were no interleaved washout epochs between stimulation at different voltages. However, despite these limitations, we think our findings are robust enough to support our conclusions.

An aspect that requires further comment is the relatively small suppression of low gamma power during effective DBS. Although significant, this represented only a 7% power reduction within this frequency band. Although this might relate to the perioperative stun effect, it should also be stressed that power in this region will have been near the noise floor of our recording setup. This may prohibit a true scalar appreciation of the DBS‐induced suppression of physiological gamma activity, as a significant proportion of the power in this band may have been due to a baseline elevated by electrical noise. Nevertheless, low gamma suppression was still very small when considered in absolute terms, less than a 3 μV reduction in signal amplitude integrated over the band, and this may prove too low for closed‐loop recording and stimulation systems focusing on tremor, without further improvement in amplifier design.

Whether low gamma activity in the region of the STN is directly related to tremor generation or represents a permissive state for tremor generation remains uncertain. Against a direct relationship between low gamma activity in the region of the STN and tremor is the fact that gamma activity (unlike beta activity) increases in power following treatment with levodopa 6, and yet levodopa can also attenuate tremor. Nevertheless, levodopa generally increases gamma at higher frequencies than the low gamma seen here, and it may also be true that there are multiple functional gamma activities in the subthalamic region, only one of which is directly or indirectly related to rest tremor. Consistent with this interpretation, low gamma activity in the STN also increases during mental stress 32, 33, when PD rest tremor also increases in amplitude 34.

Although beta activity tended to be suppressed by DBS in this cohort, this did not remain significant after correcting for the multiple ANOVAs. This is in contrast to the results of previous studies that demonstrated clear beta suppression during DBS 16, 35. This may relate to methodological differences, such as differences in the noise floor of the recording systems or frequency band selection, or phenotypic differences between the studies. Previous studies have not selected patients, whereas here we only assessed those with tremor both pre‐ and postoperatively. Finally, the beta suppression induced by DBS in this cohort may have been somewhat masked by the suppression of tremor, as prominent tremor has been associated with the suppression of beta activity in the STN and motor thalamus 36, 37, and so the converse, increase of beta activity, might be expected during tremor suppression.

In conclusion, the findings presented here strengthen the relationship between low gamma activity and rest tremor, and add to the growing evidence that different spectral elements in the STN LFP may be associated with different features of Parkinsonism. The potential clinical relevance of this lies in the prospect of individualized DBS, whereby different LFP features are tracked to guide stimulation that is most appropriate for the particular set of symptoms experienced by a given patient at that moment in time 38.

## Authorship Statement

Prof. Brown designed the study and Dr. Pogosyan was responsible for the software required for the study. Drs. Beudel, Little, and Thevathasan conducted the study; Dr. Foltynie, Prof. Limousin, Prof. Ashkan, Mr. Zrinzo, Prof. Hariz, Dr. Bogdanovic, Dr. Cheeran, Mr. Green, and Prof. Aziz were involved in patient recruitment. Drs. Beudel and Little, and Prof. Brown analyzed the data. Dr. Beudel prepared the manuscript draft with important intellectual input from Prof. Brown and Dr. Little. All authors reviewed the manuscript critically. All authors approved the final manuscript.
